# Quantifying the sensitivity limit of ICG imaging in the presence of tissue autofluorescence

**DOI:** 10.1117/1.JBO.31.5.056001

**Published:** 2026-05-04

**Authors:** Yuriy Belozerov, Jorge Ripoll, Ilya Turchin

**Affiliations:** a Federal Research Center A.V. Gaponov-Grekhov Institute of Applied Physics of the Russian Academy of Sciences, Nizhny Novgorod, Russia; b Universidad Carlos III de Madrid, Departamento de Bioingeniería, Madrid, Spain; c Instituto de Investigación Sanitaria Gregorio Marañón, Madrid, Spain

**Keywords:** fluorescence imaging, indocyanine green, autofluorescence, sensitivity, Monte Carlo method, biological tissues

## Abstract

**Significance:**

Fluorescent imaging (FI) using indocyanine green (ICG) is a powerful tool in medical diagnostics and surgery. Although numerous studies have focused on optimizing injection protocols and suppressing excitation light leakage, tissue autofluorescence has not been widely recognized as a fundamental factor limiting sensitivity.

**Aim:**

We aim to quantitatively determine the sensitivity limit for ICG detection in biological tissues, accounting for background signals from both scattered excitation light and tissue autofluorescence.

**Approach:**

We combine experiments on tissue phantoms with varying ICG concentrations and Monte Carlo numerical simulation of light transport in media with different optical properties. Human skin autofluorescence was quantified *in vivo* using a nonfluorescent reference and a model medium with a known ICG concentration.

**Results:**

It was established that skin autofluorescence is the dominant source of background, exceeding the scattered light by 4 to 25 times in the imaging system used. The determined ultimate sensitivity for ICG detection in biological tissue is $8\times 10^{-12}$ to $3\times 10^{-11}\;M$ when accounting for the autofluorescence signal.

**Conclusions:**

Tissue autofluorescence is a fundamental factor limiting the sensitivity of ICG FI in the near-infrared range. The developed approach will allow for future optimization of imaging equipment and protocols for ICG and other contrast agents.

## 1 Introduction

Fluorescent imaging (FI) with exogenous dyes is an effective method for assessing blood supply to organs and tissues, identifying tumor boundaries, detecting sentinel lymph nodes, and performing other important tasks in biology and medicine.^1^^–^^3^ The growing number of publications focused on the use of indocyanine green (ICG) in both research and clinical practice highlights the strong interest in this contrast agent and its application across various types of equipment,^2^^,^^4^^,^^5^ including topical use^5^ and endoscopy.^6^^,^^7^ The interest in ICG is due to several factors: (1) its spectral properties—the excitation and fluorescence peaks lie in the region of minimal light absorption by tissues, thereby providing high imaging depth (the reduced scattering coefficient is monotonically decreasing with wavelength function, whereas absorption coefficient has a pronounced minimum in the range ∼750 to 50 nm, and the overall attenuation has a minimum in the same wavelength region^8^); (2) the possibility of clinical use of the drug, due to its relative safety for intravenous administration; and (3) the large volume of accumulated clinical data in various fields of clinical medicine. In such applications, both static (at maximum accumulation) and dynamic monitoring of drug accumulation and elimination can be used.^9^

The quality of FI and the practical applicability of the corresponding method are largely determined by the contrast level.^10^ This contrast depends on a combination of factors: the optical properties of the contrast agent, the characteristics of the target structure (such as size, depth, and location), the ratio of accumulation between the target and the surrounding tissues, and the performance of the fluorescence imaging system itself. Image contrast is fundamentally constrained by optical background signals arising from tissue autofluorescence (AF) and backscattered excitation light leaked through emission filter. Even with no contrast agent accumulation in the surrounding tissue, these optical backgrounds remain, limiting target distinction. This limitation is quantified by the signal-to-background ratio (SBR), defined as the ratio of the fluorescent intensity to the background radiation intensity detected by the system after spectral filtration.

The literature includes several studies that suggest minimizing excitation light leakage through emission filters. For instance, study by Zhu et al.^11^ proposed a radiation collimation system designed to minimize the angular scatter incident on the interference emission filter. This approach addresses the fact that the interference filter spectral characteristics depend on the angle of incidence, which can degrade spectral selectivity. An alternative method to mitigate this issue is described by Sasagawa et al.,^12^ who employed a hybrid emission filter combining an interference filter and an absorption filter to achieve an excitation rejection ratio of $10^{8}$ at 450 nm using green fluorescent protein. The same approach was applied in diffuse fluorescence tomography with red fluorescent proteins as a contrast agent.^13^ Although that works utilized a different fluorophore and spectral range than those relevant for ICG imaging, the fundamental challenges of suppressing excitation leakage and filter autofluorescence to enhance SBR remain directly analogous. Another publication^14^ considers the use of notch and excitation filters to enhance SBR. In addition, many studies focus on optimizing the dosage of contrast agents and analyzing their accumulation dynamics.^15^ Another noteworthy contribution is the research on the synthesis and optimization of a nanocomplex based on albumin and ICG, aimed at increasing the quantum yield of ICG.^16^

A significant number of studies focus on modeling the fluorescence signal in biological tissues.^17^^,^^18^ For example, a recent publication^19^ investigated how the relative change in the fluorescent signal varies with ICG concentration, vessel depth, and other medium parameters. However, examining relative changes alone does not reveal the minimum detectable concentration, nor does it account for a crucial factor that limits SBR in *in vivo* conditions—the autofluorescence of biological tissues.

To the best of the authors’ knowledge, there are currently no studies in the literature that evaluate the ultimate sensitivity of ICG detection. This evaluation should take into account both the properties of the optical system and the contribution of autofluorescence to the background signal formation.

To determine the ultimate detectable concentration of ICG, an experimental study was conducted on model media containing ICG at various concentrations, and measurements of human skin autofluorescence in the NIR-I region were obtained. To extend these results to tissues with different optical properties, numerical simulation was used to model the ratio of backscattered excitation light to fluorescent emission across a wide range of scattering and absorption coefficients.

This work also suggests a method that separates scattered and autofluorescence background components utilizing a nonfluorescent medium. It provides a quantitative assessment of the actual autofluorescence signal level from biological tissue, calibrated against the signal level from nanomolar concentrations of a contrast agent. This approach enabled to define the characteristic order of magnitude for the optical density of emission filters for autofluorescence visualization in NIR-I.

The obtained results will help refine the design of FI systems using ICG, optimize the dosage of the contrast agent, and allow for the formulation of practical guidelines for systems designed for the autofluorescence imaging in biological tissues.

## 2 Materials and Methods

### 2.1 Model of Signal-to-Background Ratio on Fluorescence Image

SBR in ICG imaging, can be expressed as the ratio of the signal level, $S_{r}$, registered by a digital camera (or other optical detector), to the background level $S_{\mathrm{bg}}$:

$$\mathrm{SBR}=\frac{S_{r}}{S_{\mathrm{bg}}}-1.$$ (1)

The registered signal $S_{r}$. contains to components: the useful fluorescent signal, $S_{f}$, and the background radiation, $S_{\mathrm{bg}}$. This equation was used, for example, in work^20^ to illustrate the quality of ICG imaging. The background level $S_{\mathrm{bg}}$ represents the sum of theissue autofluorescence signal $S_{\mathrm{af}}$ and the signal caused by the leakage of excitation radiation scattered by tissue through the emission filter, $S_{\mathrm{sc}}$. It should be noted that the definition of SBR assumes the absence of ICG in the background tissue. Taking into account the introduced notations, the SBR can be expressed as follows:

$$\mathrm{SBR}=\frac{1}{S_{\mathrm{af}}/S_{f}+S_{\mathrm{sc}}/S_{f}}.$$ (2)

The minimum detectable concentration of ICG will be defined as the value $N_{\min}$ at which the SBR = 1, since then the area containing ICG will be twice as bright as the background, which is sufficient for identifying the ICG accumulation area. Epi-illumination technique suggests the excitation radiation power densities are far below the threshold required to induce nonlinear effects. Therefore, we assume that ICG fluorescence intensity, autofluorescence, and scattered radiation intensity are linearly dependent on the excitation radiation intensity. Consequently, $S_{\mathrm{af}}/S_{f}$ and $S_{\mathrm{sc}}/S_{f}$ in expression Eq. (2) are independent of excitation radiation intensity.

### 2.2 Methodology of Experimental Studies and Numerical Simulations

As introduced in Eq. (2), the SBR is determined by two components: $S_{\mathrm{af}}/S_{f}$ and $S_{\mathrm{sc}}/S_{f}$. The ratio of the backscattered signal to the fluorescence signal, $S_{\mathrm{sc}}/S_{f}$, is calculated using the Monte Carlo simulation for a medium with arbitrary optical properties and verified experimentally using model media. The ratio $S_{\mathrm{af}}/S_{f}$ is derived from experimental measurements of tissue autofluorescence and fluorescence from a reference model medium and then recalculated using Monte Carlo simulations of the fluorescence signal from both biological tissue and the reference medium. Below, we describe the methodology for obtaining both ratios.

To determine, the autofluorescence value, an image of the biological tissue without ICG is acquired under excitation light. The measured signal (pixel intensity averaged over ROI), $S_{\mathrm{bg}}$, represents the sum of the autofluorescence component $S_{\mathrm{af}}$ and the backscattered component $S_{\mathrm{sc}}$, that leaks through the emission filter. These components may be comparable and must be separated. To do so, we employ, a nonfluorescing model medium—polytetrafluoroethylene (PTFE). The signal from the PTFE therefore arises solely from backscattered radiation leaked through the emission filters, i.e., $S_{\mathrm{bg}}^{\mathrm{PTFE}}=S_{\mathrm{sc}}^{\mathrm{PTFE}}$. The backscattered component from the skin, $S_{\mathrm{sc}}$, is related to that of the PTFE medium, $S_{\mathrm{sc}}^{\mathrm{PTFE}}$, in the same proportion as their backscattered coefficients, $R_{d}/R_{d}^{\mathrm{PTFE}}$. The latter is measured experimentally on the same setup but without emission filters, where autofluorescence (or fluorescence) is negligible compared with backscattering. Thus, using the following equation:

$$S_{\mathrm{sc}}=\frac{R_{d}}{R_{d}^{\mathrm{PTFE}}}S_{\mathrm{bg}}^{\mathrm{PTFE}},$$ (3)

the backscattered component $S_{\mathrm{sc}}$ for the biological tissue can be found. The autofluorescence signal is then obtained by subtracting the scattered component Eq. (3) from the total background:

$$S_{\mathrm{af}}=S_{\mathrm{bg}}-S_{\mathrm{sc}}.$$ (4)

To determine $S_{\mathrm{af}}/S_{f}$, the fluorescence signal $S_{f}$ should be measured under the same experimental conditions (exposure time, laser power, lens magnification and aperture stop) as $S_{\mathrm{af}}$. However, direct measurement of $S_{f}$ from ICG introduced into human tissue would require invasive procedures and ethical approval and is complicated by the unknown concentration of ICG in the tissue. Therefore, an indirect approach was adopted. Accordingly, fluorescence signal $S_{f0}$ from a reference medium—a Lipofundin solution with defined optical properties and ICG concentration—and the autofluorescence signal $S_{\mathrm{af}}$ of biological tissue were measured under the same experimental conditions. Subsequently, Monte Carlo simulations of the fluorescence and background signals were carried out over a wide range of optical parameters, as detailed in Sec. 2.6. By combining the simulation results with the measured $S_{f0}$ from the reference medium, the expected fluorescence signal $S_{f}$ for biological tissue was also calculated and consequently the ratio $S_{\mathrm{af}}/S_{f}$. Within the same simulation framework, the ratio $S_{\mathrm{sc}}/S_{f}$ for biological tissues was also obtained.

Furthermore, the developed Monte Carlo model was verified against the experimentally determined $S_{\mathrm{sc}}/S_{f}$ ratio for Lipofundin. Because Lipofundin exhibits weak autofluorescence, the background separation method described above with the help of PTFE medium and Eqs. (3) and (4) was also applied in this verification.

All key notations are summarized in Table 1.

**Table 1:** List of key notations.

Parameter	Definition
SBR	Signal-to-background ratio. Calculated by Eq. (1)
$S_{\mathrm{bg}}$	Signal from the object of study without ICG (human skin or Lipofundin solution without ICG). Measured experimentally
$S_{r}$	Signal from the object of study with ICG (Lipofundin solution with ICG). Measured experimentally
$S_{f}$	Fluorescent signal caused by the presence of ICG in the object of study. Calculated from experimental data for Lipofundin solution or simulated by MC for biological tissue.
$S_{\mathrm{af}}$	Autofluorescent signal from the object of study (human skin or Lipofundin). Calculated by Eq. (4)
$S_{f0}$	Fluorescent signal from Lipofundin solution with the basic ICG concentration ($\mu_{a0}=0.02\;{\mathrm{cm}}^{-1}$, $\mu_{s0}^{\prime }=20\;{\mathrm{cm}}^{-1}$, $n_{0}=6.5\times 10^{-10}\;M$). Calculated from experimental
$S_{\mathrm{sc}}$	Backscattered signal from the object of study (human skin or Lipofundin). Calculated by Eq. (3)
$\frac{R_{d}}{R_{d}^{\mathrm{PTFE}}}$	The ratio of the diffuse reflectance coefficients for the object of study and PTFE measured in the absence of emission filters. Measured experimentally
$S_{\mathrm{bg}}^{\mathrm{PTFE}}$	Signal $S_{\mathrm{bg}}$ from PTFE. Measured experimentally

### 2.3 Experimental Setup for Fluorescent Imaging

**Fig. 1:**
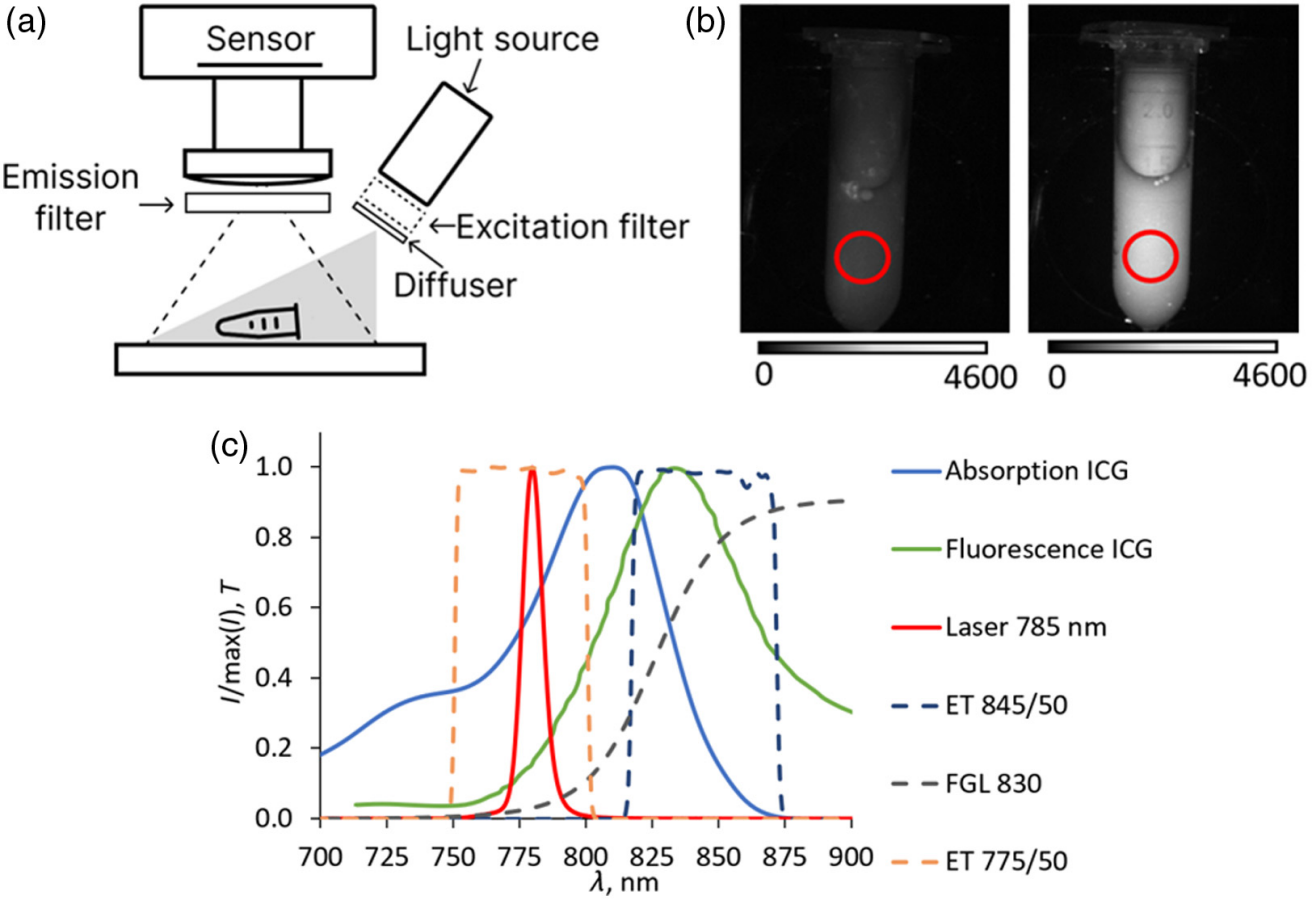
(a) Schematic of the experimental setup for fluorescent imaging; (b) experimentally obtained images of a Lipofundin model medium without ICG (left) and with added ICG at a concentration of $6.5\times 10^{-11}\;M$ (right), the ROIs used for calculating the average S signals are highlighted in red; (c) spectral characteristics of ICG absorption and fluorescence for blood plasma, taken from Ref. 21, and the used filters and radiation sources.

For the experimental study of FI SBR using ICG, an experimental setup shown in Fig. 1(a) was assembled. An ICG-containing sample is irradiated with excitation light at 785 nm generated by a laser diode (Wuhan CS Tec Co, Ltd, China) with a fiber output equipped with an ET 775/50 excitation filter (Chroma Technology, USA). An optical diffuser ED1-S20 (Thorlabs, USA) is used to provide uniform illumination of the sample. The power density on the sample was $20\;\mathrm{mW}/{\mathrm{cm}}^{2}$. The plane of laser illumination coincides with the focal plane of the camera lens. The emission filter consisted of a combination of an interference filter (IF) ET845/50 (Chroma Technology, USA) with an optical density OD = 8 at the excitation wavelength and an absorption filter FGL830 (Thorlabs, USA) with OD = 1.2 at the excitation wavelength. A monochrome camera a2A2840-48umBAS (Basler, Germany) with an MTV12MP5C (ZLKC, China) lens is used for image acquisition. The spectral characteristics of the filters and light sources are shown in Fig. 1(c). To minimize the own noise of the camera, all measured signals were averaged over the region of interest shown in Fig. 1(b). Exposure time was 5 to 10 s, depending on the signal level.

This experimental setup was also used to obtain images of finger skin. To analyze the dependence of autofluorescence properties on the excitation wavelength, the following additional laser diodes with fiber output were used: 650 nm (Hicurtec, Russia), 690 nm (Hicurtec, Russia), 760 nm (Wuhan CS Tec Co, Ltd, China), and 792 nm (BWT, China).

### 2.4 Experimental Evaluation of the Scattered Component’s Contribution to the Background of Lipofundin Solution

An experimental study of the $S_{\mathrm{sc}}/S_{f}$ value was performed on the FI setup using model media. The model fluorescent medium was a 20% Lipofundin solution (B. Braun, Germany) with ICG (PULSION MEDICAL SYSTEMS, Germany) added at concentrations of $6.5\times 10^{-7}$, $6.5\times 10^{-8}$, $6.5\times 10^{-9}$, and $6.5\times 10^{-10}\;M$. The solution was placed in a 2-mL laboratory cuvette. The same Lipofundin solution without ICG served as the background model. The absorption and reduced scattering coefficients^22^ of this solution are $\mu_{a0}=0.02\;{\mathrm{cm}}^{-1}$ and $\mu_{s0}^{\prime }=20\;{\mathrm{cm}}^{-1}$.

The value of $S_{f}$ was calculated as the difference between the signals from the cuvette containing ICG and the cuvette containing only Lipofundin, averaged over identical ROIs in the images [Fig. 1(b)].

Because Lipofundin may exhibit weak autofluorescence, the value of $S_{\mathrm{sc}}$, caused by light scattering, can be estimated according to Eq. (3) by normalizing to the signal from a nonfluorescent PTFE medium, $S_{\mathrm{bg}}^{\mathrm{PTFE}}$, which includes only the scattered component. The ratio of backscattered coefficients of Lipofundin and PTFE media $R_{d}/R_{d}^{\mathrm{PTFE}}$ was measured on the same experimental setup without emission filters.

### 2.5 Experimental Evaluation of the Autofluorescence Component’s Contribution to the Background of Biological Tissues

To date, the sources of autofluorescence in biological tissues in the near-infrared wavelength range remain poorly studied^23^; therefore, the $S_{\mathrm{af}}$ value was determined experimentally. The skin of the index finger of one of the authors was used to estimate this parameter. The experiments were performed on the setup described in Sec. 2.3, using the following excitation wavelengths: 650, 690, 760, 785, and 792 nm.

Because the level of autofluorescence in the near-infrared range is quite weak, the scattered component $S_{\mathrm{sc}}$, which may be comparable to it, must be subtracted from the measured background signal $S_{\mathrm{bg}}$ according to Eq. (4). The $S_{\mathrm{bg}}$ value is obtained by averaging over the ROI on the recorded image. The $S_{\mathrm{sc}}$ component can be accounted for using Eq. (3).

To standardize the measured autofluorescence signal $S_{\mathrm{af}}$, it was normalized to the fluorescence signal from a reference medium, $S_{f0}$, with a known ICG concentration, following the approach outlined in Sec. 2.2. For this purpose, the Lipofundin solution described in Sec. 2.4, with an ICG concentration of $n_{0}=6.5\times 10^{-10}\;M$ was chosen. By combining this normalized value, $S_{\mathrm{af}}/S_{f0}$, with the results of Monte Carlo simulations, one can then calculate the ratio $S_{\mathrm{af}}/S_{f}$ for biological tissue with arbitrary optical properties, as described in Sec. 2.6.

### 2.6 Monte-Carlo Model of SBR in Biological Tissues

A numerical model based on Monte Carlo (MC) simulation was developed to verify the experimental results obtained on model medium and evaluate the SBR within biological tissues. This model simulates a semi-infinite, homogeneously scattering, absorbing, and fluorescing medium illuminated by a plane wave at the excitation wavelength. The MC simulated three key quantities: the diffuse reflection coefficient $R_{d}$ (the ratio of the escaping flux density to the incident irradiance), the relative fluorescence level $R_{f}$ (the ratio of the escaping fluorescent flux density to the incident irradiance), and their ratio $R_{f}/R_{d}$.

The MC model was developed based on the source code created by Steve Jacques and described in the paper,^24^ implemented in a cylindrical coordinate system. The simulation was conducted in three consecutive stages.

In the first stage, the spatial distributions of absorbed excitation light energy in the tissue and of backscattered photons were calculated. This stage accounted for absorption and scattering in the sample and reflection of radiation at the medium boundary. The optical properties of the medium were varied within the following ranges: absorption coefficient $\mu_{a}\in [0.01,0.02,0.04,0.08,0.16,0.32,0.54,1.28]\;{\mathrm{cm}}^{-1}$, transport scattering coefficient $\mu_{s}^{\prime }\in [5,10,20,40,80,160]\;{\mathrm{cm}}^{-1}$, which amply covers the typical range of these values for biological tissues in the near-infrared range,^25^ as well as the optical parameters of the model medium. The scattering anisotropy coefficient was set to 0.9. The dimensions of the simulated object were 4 cm in depth and 2 cm in the radial direction, exceeding the maximum photon transport mean free path by 20 and 10 times, respectively. For each pair of scattering and absorption coefficients, $10^{7}\;\text{photons}$ were launched.

The second stage simulated fluorescence from a uniformly distributed fluorophore. This was modeled by treating each cell in the computational grid as an isotropic point source. The intensity of each source was proportional to the number of absorbed photons in that cell and the fluorophore’s specific properties: its concentration, molar extinction coefficient, and quantum yield. This product was also normalized by the medium’s absorption coefficient. The data for ICG corresponded to the parameters of the model medium at a wavelength of 785 nm: concentration, $n_{0}=6.5\times 10^{-10}\;M$, molar extinction coefficient, $\varepsilon_{0}=180,000\;M^{-1}\cdot {\mathrm{cm}}^{-1}$, quantum yield, ${\mathrm{QY}}_{0}=0.02$.^26^^–^^29^ The scattering and absorption coefficients of the medium for fluorescent radiation were assumed to be the same as for the excitation radiation.

In the final stage, the values of $R_{d}$ and $R_{f}$, and $R_{f}/R_{d}$ were computed. Specifically, $R_{d}(\mu_{a},\mu_{s}^{\prime })$ was determined for each $\left(\mu_{a},\mu_{s}^{\prime }\right)$ pair as the ratio of the weighted photon density escaping the medium at the excitation wavelength to the incident photon density. To minimize edge effects, the calculation considered only photons exiting from a central platform with a radius of 1.2 cm.

The relative fluorescence level $R_{f}$ was calculated similarly for the density of fluorescent photons escaping the medium.

We assume that ICG concentration is low enough to neglect the self-quenching effects^30^ and the absorption due to ICG compared with that of the biological tissue. Thus, to calculate $R_{f}$ value in tissues with an arbitrary ICG concentration—n, molar extinction coefficient—ε, and quantum yield—QY, a linear dependence of fluorescence intensity on these quantities was used:

$$R_{f}(\mu_{a},{\mu^{\prime }}_{s},n,\varepsilon ,\mathrm{QY})=\frac{n\varepsilon QY}{n_{0}\varepsilon_{0}QY_{0}}R_{f}(\mu_{a},{\mu^{\prime }}_{s},n_{0},\varepsilon_{0},{\mathrm{QY}}_{0}).$$ (5)

To estimate the variation range of the $R_{f}$ value in tissues, the following ranges of these parameters were used: the molar extinction coefficient,^26^^,^^27^ ε, was 150,000 to $220,000\;M^{-1}\cdot {\mathrm{cm}}^{-1}$; the fluorescence quantum yield, QY, was 0.12 to 0.14 when diluted in whole blood^25^ and 0.02 to 0.03 in Lipofundin.^26^^–^^29^ Similarly, the obtained results were extrapolated to concentrations, n, which varied from $6.5\times 10^{-10}$ to $6.5\times 10^{-7}\;M$.

To compare MC simulation results with the results of the model experiment and, accordingly, to account for the $S_{\mathrm{sc}}/S_{f}$ value in the SBR model Eq. (2), it is necessary to consider the spectral filtration of the experimental setup (implemented by the emission filter system). Taking spectral filtration into account, the relationship between the simulated ratio $R_{f}/R_{d}$ and the experimentally measured $S_{\mathrm{sc}}/S_{f}$ ratio can be expressed as

$$\frac{S_{\mathrm{sc}}}{S_{f}}=\frac{R_{d}}{R_{f}}\frac{T_{\mathrm{ex}}}{T_{\mathrm{em}}},$$ (6)

where $T_{\mathrm{em}}$ is the transmission coefficient of the emission filter in the spectral range of fluorescence and $T_{\mathrm{ex}}$ is the transmission coefficient of the emission filter in the spectral range of the excitation radiation. In the experimental setup described in Sec. 2.3: $T_{\mathrm{em}}=0.28$ and $T_{\mathrm{ex}}=10^{-9.2}$.

Accounting for the autofluorescence component $S_{\mathrm{af}}/S_{f}$ in the SBR model Eq. (2) for a medium with arbitrary scattering, absorption, ICG concentration, and its photometric parameters can be achieved using the experimentally obtained ratio—$S_{\mathrm{af}}/S_{f0}$ (Sec. 2.5). The transformation is performed by recalculating $S_{f0}=S_{f0}(\mu_{a0},\mu_{s0}^{\prime },n_{0},\varepsilon_{0},{\mathrm{QY}}_{0})$ into $S_{f}(\mu_{a},\mu_{s}^{\prime },n,\varepsilon ,\mathrm{QY})$, using the results of the MC simulation for the relative fluorescence level $R_{f}$

$$\frac{S_{\mathrm{af}}}{S_{f}}=\frac{S_{\mathrm{af}}}{S_{f0}}\frac{R_{f}(\mu_{a0},\mu_{s0}^{\prime },n_{0},\varepsilon_{0},{\mathrm{QY}}_{0})}{R_{f}(\mu_{a},\mu_{s}^{\prime },n,\varepsilon ,\mathrm{QY})}.$$ (7)

## 3 Results

### 3.1 Investigation of the Autofluorescence Contribution to the Background

**Fig. 2:**
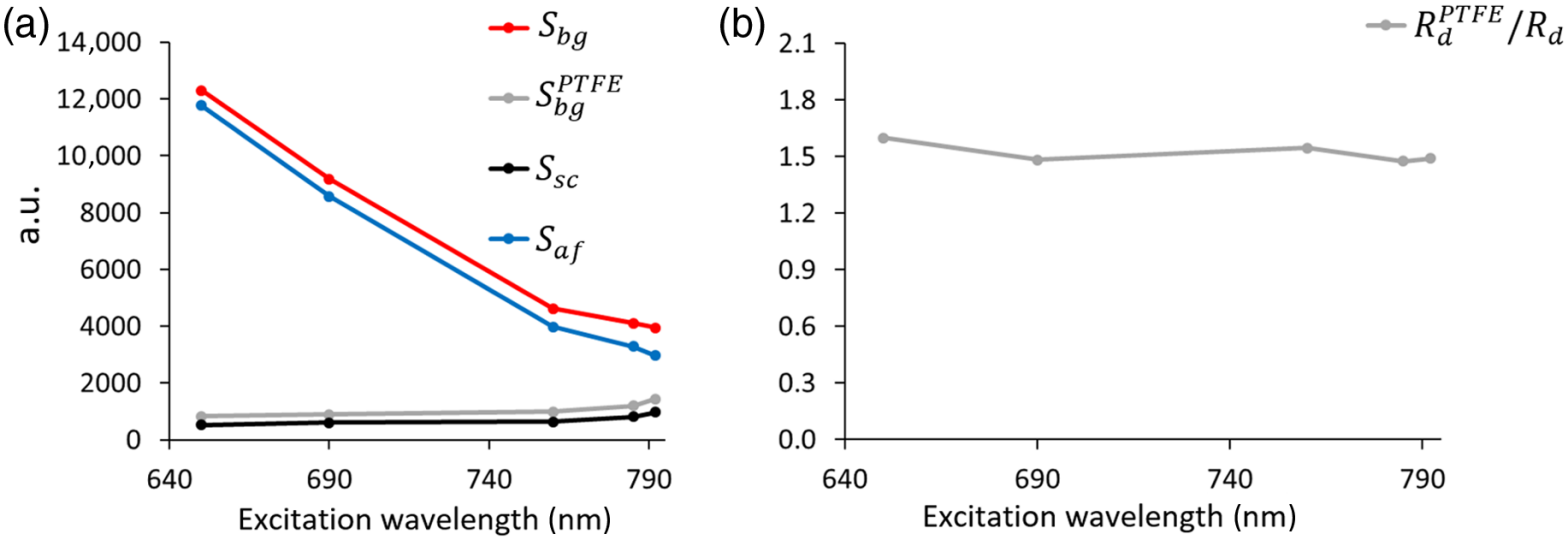
(a) Experimental dependences of the registered signal level on the excitation wavelength for finger skin $S_{\mathrm{bg}}$ and PTFE medium $S_{\mathrm{bg}}^{\mathrm{PTFE}}$; the separated components $S_{\mathrm{af}}$ and $S_{\mathrm{sc}}$ responsible for autofluorescence and scattering of the skin, respectively, are also shown; (b) measured ratio of the diffuse reflection coefficients of finger skin and PTFE medium $R_{d}^{\mathrm{PTFE}}/R_{d}$.

Figure 2(a) presents the signals from the finger skin $S_{\mathrm{bg}}$ and from PTFE medium $S_{\mathrm{bg}}^{\mathrm{PTFE}}$ measured with the experimental fluorescence imaging setup, showing their dependence on the central wavelength of the excitation source. The measured ratio of the diffuse reflection coefficients $R_{d}^{fl}/R_{d}$, required to account for the scattered background component according to Eq. (4), is presented in Fig. 2(b).

As shown in the Fig. 2(b), this ratio is independent of the wavelength within the studied range. The value of $S_{\mathrm{af}}$ calculated using Eq. (4) is presented in Fig. 2(a) (blue line). The data show that $S_{\mathrm{af}}$ increases as the excitation wavelength decreases. The obtained dependence can be interpreted as a fragment of the absorption spectrum of endogenous fluorophores that determine the autofluorescence properties of finger skin.

As shown in Fig. 2(a), the autofluorescence signal from the finger skin significantly exceeds the contribution of the scattered component. At an excitation wavelength of 792 nm, the autofluorescence intensity is 4 times higher than the backscattered component $S_{\mathrm{sc}}$. At shorter wavelengths, this ratio increases, reaching 25. Within this experiment, the ratio $S_{af}/S_{f0}=0.16$ was also obtained, where the signal $S_{\mathrm{af}}$ was 3200 a.u. [Fig. 2(a), 785 nm], and $S_{f0}$ for a concentration of $n_{0}=6.5\times 10^{-10}\;M$ was 20,000 a.u. The number of pixels in the ROI was 20,050, the instrumental noise was about 3.5% of the minimum measured value, which can be considered negligible for the purpose of this study.

### 3.2 Determination of the Minimum Detectable Concentration in Biological Tissue

**Fig. 3:**
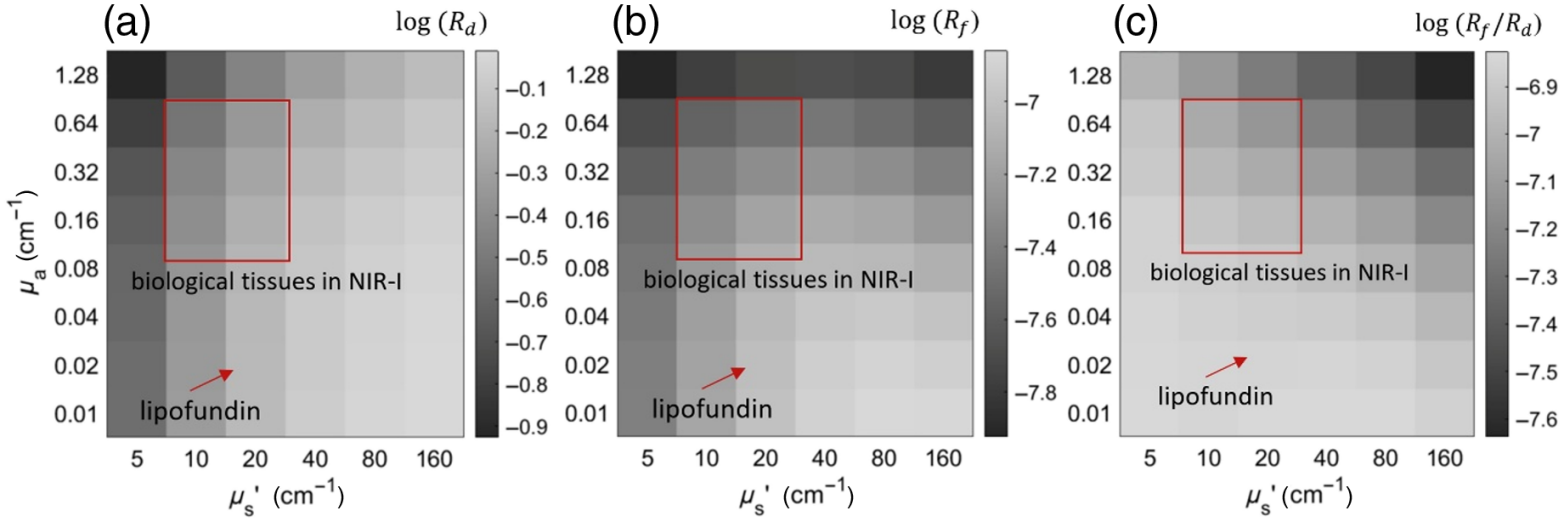
(a) Matrix of logarithms of the medium’s diffuse reflection coefficient $R_{d}$; (b) matrix of logarithms of the relative fluorescence level, $R_{f}$; (c) matrix of logarithms of the ratio of $R_{f}$ to $R_{d}$. The data are presented for various absorption coefficients $\mu_{a}$ and reduced scattering coefficients $\mu_{s}^{\prime }$. The region associated with the optical parameters of biological tissues in the near-infrared range is indicated by a red rectangle. The calculation results are presented for an ICG concentration in the medium of $n_{0}=6.5\times 10^{-10}\;M$, a molar extinction coefficient of $\varepsilon_{0}=180,000\;M^{-1}\cdot {\mathrm{cm}}^{-1}$, and a fluorescence quantum yield of ${\mathrm{QY}}_{0}=0.02$.

Figure 3 presents the results of calculating the values $R_{d}$, $R_{f}$, and $R_{f}/R_{d}$ for a medium with absorption and scattering coefficients in the ranges $\mu_{a}\in (0.01\;\text{to}\;1.28)\;{\mathrm{cm}}^{-1}$, $\mu_{s}^{\prime }\in (5\;\text{to}\;160)\;{\mathrm{cm}}^{-1}$ at an ICG concentration in the medium of $n_{0}=6.5\times 10^{-10}\;M$, a molar extinction coefficient of $\varepsilon_{0}=180,000\;M^{-1}\cdot {\mathrm{cm}}^{-1}$, and a fluorescence quantum yield of ${\mathrm{QY}}_{0}=0.02$. For the model medium—a Lipofundin solution containing ICG at concentration $n_{0}$—the calculated $R_{f}/R_{d}$ value was $1.42\times 10^{-7}$ [Fig. 3(c)]. Using this data, the $S_{\mathrm{sc}}/S_{f}$ ratio was estimated according to Eq. (6).

For the optical parameters of biological tissues, whose value range is indicated by the red rectangle in Fig. 3, the $R_{f}/R_{d}$ value for a concentration of $n_{0}$ varies within a narrow range: $5.2\times 10^{-7}$ to $7.2\times 10^{-7}$, and the range of values for the $S_{sc}/S_{f}$ quantity was 0.003 to 0.004.

**Fig. 4:**
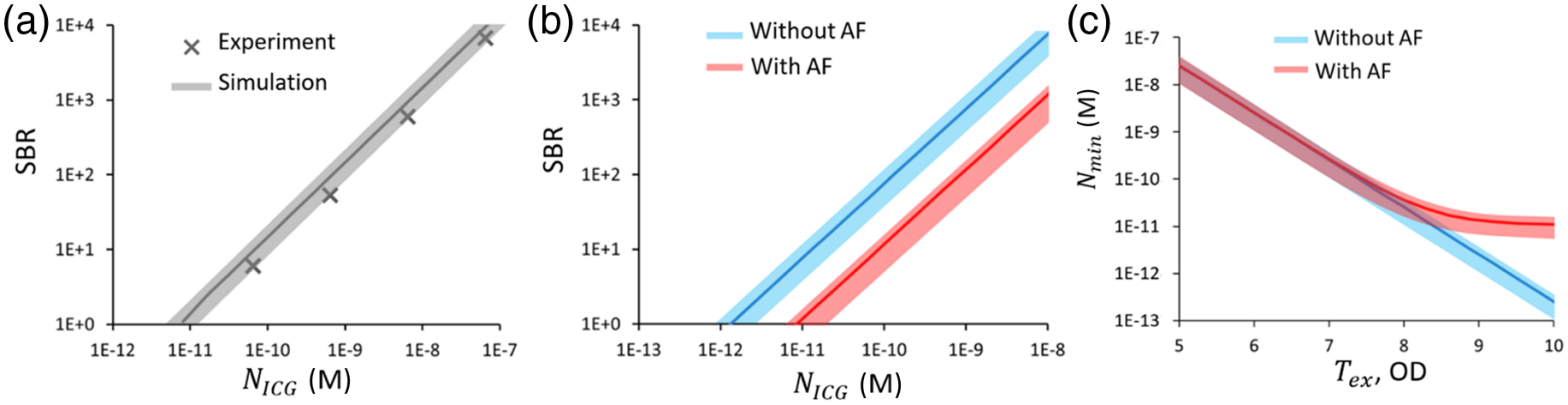
(a) Experimental points (’x’ markers) and simulated (solid lines) dependences of SBR on ICG concentration in Lipofundin, (b) simulated dependences of contrast on ICG concentration in biological tissue, and (c) simulated minimum detectable concentration in biological tissue depending on the optical density of the emission filter. Model parameters accounting for with AF (red lines) and without AF (blue lines): ICG concentration $N_{\mathrm{ICG}}=6.5\times 10^{-11}$ to $6.5\times 10^{-8}\;M$, molar extinction coefficient $M=180,000\;M^{-1}\cdot {\mathrm{cm}}^{-1}$, fluorescence quantum yield QY is 0.025 for Lipofundin and 0.13 for biological tissue, optical parameters $\mu_{a0}=0.02\;{\mathrm{cm}}^{-1}$ and $\mu_{s0}^{\prime }=20\;{\mathrm{cm}}^{-1}$ for Lipofundin, and $\mu_{a}=0.40\;{\mathrm{cm}}^{-1}$ and $\mu_{s}^{\prime }=15\;{\mathrm{cm}}^{-1}$ for biological tissues. Value ranges highlight the model parameter intervals described in Sec. 2.5.

To account for the autofluorescence background, the experimental $S_{\mathrm{af}}/S_{f0}$ ratio was calculated using Eq. (7). This equation incorporates the ratio $R_{f}(\mu_{a0},\mu_{s0}^{\prime },n_{0},\varepsilon_{0},{\mathrm{QY}}_{0})/R_{f}(\mu_{a},\mu_{s}^{\prime },n,\varepsilon ,\mathrm{QY})$, which was derived from the MC modeling results presented in Fig. 3(b). The resulting values of $S_{\mathrm{af}}/S_{f}$ at a concentration $n_{0}$ and an excitation wavelength of 785 nm fell within the range between 0.009 and 0.026. The calculation of values for other concentrations was performed using Eq. (5). The results of the numerical simulation of the SBR and $N_{\min}$ for biological tissues are presented in Figs. 4(b) and 4(c). Figure 4(b) shows the dependences of SBR on ICG concentration, and Fig. 4(c) presents the minimum detectable concentration depending on the optical density of the emission filter at the excitation wavelength. The results are given for two cases: without accounting for autofluorescence (blue curves) and with its inclusion (red curves). In Fig. 4(a), it can be seen that the experimentally measured values of $S_{\mathrm{sc}}/S_{f}$ in Lipofundin fall within the range of the simulated values.

These results show that accounting for the autofluorescence signal leads to a six-fold decrease in SBR across the entire studied concentration range. The minimum detectable concentration, $N_{\min}$, for the FI system used, which has a filter optical density of OD = 9.2, is $9\times 10^{-13}$ to $3\times 10^{-12}\;M$ without accounting for autofluorescence and $8\times 10^{-12}$ to $3\times 10^{-11}\;M$ when accounting for it. Furthermore, as can be seen from the graph, a further increase in optical density will not lead to a reduction in the minimum detectable concentration, because the autofluorescence background begins to dominate over the backscattered component, and $N_{\min}$ asymptotically approaches $6\times 10^{-12}\;M$. This result qualitatively agrees with experimental observations and reflects a physically justified detection limit.

## 4 Discussion

In this work, a numerical and experimental study of the minimum detectable concentration of ICG in a scattering medium, taking into account the background associated with scattering and autofluorescence, was conducted. For the model medium, which is a mixture of Intralipid and ICG at different concentrations, the experimental results showed good agreement with the numerical simulation data [Fig. 4(a)], confirming the accuracy of the MC calculations. The simulation results for $R_{d}$ also correlate well with the model presented in Ref. 31 for a semi-infinite turbid medium, especially in the region of high values of the absorption coefficient $\mu_{a}$ and the reduced scattering coefficient $\mu_{s}^{\prime }$. The ratio $R_{f}/R_{d}$ presented in Sec. 3.2 is also equivalent to the ratio of fluorescence to backscattered photons before spectral filtering and therefore is independent of the experimental setup. The ratio $S_{\mathrm{af}}/S_{f}$ can be calculated according to Sec. 2.6 for arbitrary contrast agent in the far-red-NIR-I spectral region, taking into account its quantum yield, molar extinction coefficient, and concentration within the provided range of optical parameters.

Accounting for the background component associated with autofluorescence was performed using in vivo data obtained from finger skin and MC simulation results [Figs. 4(b) and 4(c)]. This allowed the calculation of $S_{\mathrm{af}}/S_{f}$ value using Eq. (7) for biological tissue optical properties. The established sensitivity limit of $∼10^{-11}\;M$ is comparable to the data from the most sensitive molecular imaging methods, such as PET.^32^

Direct *in vivo* validation was precluded by the fundamental difficulty of establishing the true ICG concentration in tissues, as it continuously evolves following intravenous injection. Whole blood proves unsuitable as a reference medium due to erythrocyte sedimentation. Lipofundin solution with ICG was employed for the experiments as a well-controlled experimental medium which can be easily reproduced by other researchers. Crucially, Lipofundin served not as a tissue model phantom, but as a reference medium. The reference signal from Lipofundin was thus transformed into tissue-specific values via MC simulations data.

The simulation was performed for an optically homogeneous scattering and absorbing medium containing a uniformly distributed contrast agent. This assumption represents a significant simplification compared with real biological structures, which creates a highly inhomogeneous spatial distribution. Any additional factors in a real biological environment, such as target depth and finite size cause higher attenuation of the fluorescent signal, while not affecting the level of the background signal, which will obviously lead to a decrease in the SBR and an increase in the minimum detectable concentration. Therefore, the value obtained in this work represents the lower limit of detectable concentration for the given fluorophore. Accounting for the realistic spatial distribution of the contrast agent can be performed using Monte Carlo simulation in a complex inhomogeneous medium, as is done, for example, in Ref. 19.

It should be noted that the presence of ICG in the surrounding tissues can significantly reduce contrast, whereas the SBR value remains unchanged because it does not account for ICG in the surrounding tissue. For tumor visualization, contrast at high ICG concentrations (near the maximum of accumulation) is governed by vascular density and extravasation, whereas at low concentrations—such as during long-term monitoring as the agent clears—autofluorescence and scattering become the limiting factors, due to low concentrations of ICG in surrounding tissues. Sentinel lymph node detection is limited primarily by tissue depth and skin autofluorescence. In anastomosis assessment, background signal associated with ICG leakage from vessels is not significant, as it occurs only in areas with normal blood circulation; here, contrast between perfused and nonperfused tissues is determined by autofluorescence and scattered light.

It should also be noted that the model did not account for the contribution of ICG absorption to the total absorption of the medium, since for concentrations on the order of $∼10^{-10}\;M$, its value ($∼1.8\times 10^{-5}\;{\mathrm{cm}}^{-1}$) is significantly lower than the absorption of biological tissues. Furthermore, the secondary absorption of fluorescent photons by fluorophore molecules was not taken into account, because according to the study by Maarek et al.,^30^ the self-quenching effect begins to manifest at an ICG concentration of $2.58\times 10^{-5}\;M$, which is four orders of magnitude higher than the assessed minimum detectable concentration. These assumptions are valid but limit the applicability of the model to the low-concentration region, as higher concentrations may lead to changes in optical properties and the manifestation of fluorescence self-quenching effects.

A notable feature is the nonmonotonic behavior of the relative fluorescence level, $R_{f}$, [Fig. 3(b)], observed with an increase in the reduced scattering coefficient under conditions of high absorption. We offer the following explanation for this effect. At low absorption, an increase in the scattering coefficient leads to a higher number of fluorescent photons because the excitation photons wander longer paths in the medium, increasing their probability of being absorbed by the fluorophore. In the case of significant absorption, an increase in scattering initially enhances fluorescence generation; however, a further increase leads to a reduction in the effective penetration depth of the excitation radiation due to intensified attenuation. This limits the volume of the medium participating in fluorescence generation, which ultimately causes a decrease in the number of fluorescent photons.

For many medical applications of FI, including sentinel lymph node identification and blood supply assessment, the skin is the primary source of the background signal. Therefore, it was selected as the biological tissue for investigation. The results demonstrate that in the experimental setup used, the level of skin autofluorescence exceeds the intensity of backscattered radiation by 4 to 25 times, depending on the excitation wavelength. Varying the excitation wavelength within the range of 650 to 792 nm was motivated by two factors: (1) the significant scientific and clinical interest in direct autofluorescence imaging of biological tissues in the NIR range and (2) the need to quantify the contribution of AF to the background in studies using other fluorophores in far-red and NIR. Regarding the first point, NIR AF has been successfully used for intraoperative identification of parathyroid glands,^33^^,^^34^ tissue viability assessment, and diagnosis of skin lesions.^33^^,^^34^ It is known that the fluorescence of endogenous fluorophores increases with decreasing wavelength in the far-red to near-infrared region,^23^ but these data have not previously been quantitatively characterized. A versatile imaging system can be configured to operate in both autofluorescence imaging and ICG imaging modes with a single light source; therefore, understanding the spectral behavior of autofluorescence is critically important. Although the change in ICG signal level with excitation wavelength can be estimated from its known absorption spectrum, the corresponding change in autofluorescence background level must be measured experimentally, as demonstrated in our study.

The observed decrease in the autofluorescence signal in the near-infrared region of the spectrum [Fig. 2(a)] indicates the promise of extending research to the NIR-II range (1000 to 1700 nm). Unlike the traditional NIR-I range, imaging in the NIR-II range is characterized by significantly reduced light scattering in biological tissues, which provides greater probing depth and higher spatial resolution. Furthermore, according to our results, transitioning to the NIR-II range potentially allows for further minimization of the influence of tissue autofluorescence, as also noted in other works.^35^^–^^37^ However, the efficiency of ICG excitation in the NIR-II range is substantially reduced compared to the traditional NIR-I window. Given the active discussion on the possibilities of ICG FI in the NIR-II range,^38^^,^^39^ applying the approach presented in this work for a comparative analysis of ICG imaging efficiency in both spectral windows would be a promising continuation of the present study.

## 5 Conclusions

This work establishes that the autofluorescence of biological tissues is the dominant factor limiting the SBR and sensitivity of FI with ICG in the near-infrared range. The experimental-numerical approach was used to determine the minimum detectable concentration of ICG under conditions close to *in vivo* at $8\times 10^{-12}$ to $3\times 10^{-11}\;M$. The results demonstrate that beyond a certain value, increasing the optical density of emission filters does not lead to a significant improvement in sensitivity, as the autofluorescence background becomes the limiting factor. This result indicates a fundamental threshold for existing imaging systems in the NIR-I range. The developed model and the obtained quantitative estimates have important practical significance for optimizing the dosage of the contrast agent and designing fluorescent equipment, allowing for the minimization of the risk of false-negative results.

## Data Availability

The data presented in this article are available on reasonable request.
